# Unraveling genetic regulatory networks of mammalian retroelements

**DOI:** 10.1186/1753-6561-3-s2-s3

**Published:** 2009-03-10

**Authors:** Kenneth S Ramos

**Affiliations:** 1 Department of Biochemistry and Molecular Biology & Center for Genetics and Molecular Medicine, University of Louisville School of Medicine, Louisville, Kentucky, USA

## Abstract

Advances in the science of toxicogenomics have opened the door to major advances in our understanding of the molecular basis of environmental pathogenesis and the role of environmental factors in human disease. This report summarizes major findings in the laboratory defining the molecular basis of L1 retroelement activation in mammalian cells and the architecture of gene regulatory networks involved in phenotypic control.

## Background

Mammalian retroelements are ubiquitous genetic elements that amplify themselves in the mammalian genome via reverse transcriptase and that modify the biology of cells by reinsertion and possibly by modulation of RNA and microRNA biology. Eukaryotic retroelements rely on reverse transcriptase to transpose via an RNA intermediate to a different location. The general structure of a "typical" retroelement includes two open reading frames and 5' and 3' untranslated regions involved in regulation of transcriptional activity and stability.

In mammals, up to 42% of the genome is believed to be comprised of retrotransposons; this translates into millions of elements most of which are not able of self replication or so called "dead". Of particular interest are members of the LINE-1 (L1) family, abundant elements in the human and mouse genomes believed to have contributed extensively to genomic evolution and to participate in reprogramming of genetic programs during the course of development and pathogenesis. L1s are targeted for epigenetic silencing during early embryonic development and remain inactive in most cells and tissues upon completion of cellular differentiation. Understanding how retrotransposons and their host genomes have coevolved and the molecular mechanisms that help to optimize mutual survival is still in its infancy. The ENCODE Project shows that protein coding DNA makes up barely 2% of the overall genome, yet 80% of 30 million bases analyzed to date show evidence of being expressed) [1].

## Methods

Within the context of environmental disease, research in my laboratory over the past 15 years has identified retroelements as molecular targets of environmental carcinogens [1-8]. The ability of genotoxic agents to reactivate mammalian retroelements has guided considerable efforts to elucidate molecular mechanisms of genetic reactivation and its implications in the onset and progression of environmental diseases of cellular growth and differentiation such as atherosclerosis and cancer. Of relevance are the possible roles of retroelements in mutagenesis (inversions, duplications and insertions) as well as DNA repair. In our studies, we have focused on the role of L1 in regulating the transition of normal and disease phenotypes. Our hypothesis is that reactivation of L1 by environmental carcinogens upsets the regulatory mechanisms involved in fixation of cellular differentiation programs to recapitulate early developmental programming and give rise to altered phenotypes characteristic of atherosclerotic vessels and cancer.

## Results

Earlier studies focused on the transfection of HeLa cells with a human L1 element tagged with a Neo cassette in the 3' untranslated region in order to evaluate the L1 mobilization and reinsertion upon challenge with low doses of benzo(a)pyrene, a widespread environmental carcinogen and atherogen. The construct used has two promoters; P1 and P2 drive transcription in opposite directions, a β-globin intron inserted into Neo cassette in opposite orientation, splicing donor and acceptor sites and G418 resistance gene [9]. The specificity of these interactions was assessed using a reverse transcriptase mutant. Under these experimental conditions, antibiotic resistance would only be achieved by activation of reverse transcription, splicing and genomic integration. G418 resistant clones were found to contain an intact Neo cassette integrated into their genome. In parallel experiments it was shown that BaP induced the synthesis of L1 cDNA in HeLa cells. Interestingly, L1 expression increased the resistance of cells to G418 mediated apoptosis, a finding consistent with the hypothesis that reactivation of L1 by environmental carcinogens mediates genetic reprogramming and modulates cellular phenotypes.

Little is understood about the complex biology of L1 elements. To begin to elucidate genetic interactions of L1 computational biology studies have been initiated to elucidate the structure of L1 gene networks and to define biological interactions of relevance to the regulation of cellular phenotypes. Given the abundance of retroelements in the mammalian genome and the extensive redundancy associated with elements that are no longer active, our initial studies focused on the study of an L1 element previously identified in the mouse genome and localized to chromosome 4 as a "unique" element [10]. The discretization of genetic networks was achieved using a Coefficient of Determination (CoD) algorithm employed in previous studies to elucidate biological networks that mediate induction of atherogenic phenotypes in cultured vascular smooth muscle cells [11]. CoD relies on Boolean logic to define binary or ternary functions that describe genetic interactions within a biological system. DNA microarray experiments from the public domain database were employed to identify predictors of L1 as a target. The results of these analyses identified a member of the PAS homology domain superfamily as a central node within the predicted L1 regulatory network. Given that other members of this superfamily, namely the aryl hydrocarbon receptor (AHR), had been previously identified as regulators of L1 activity, biological experiments were carried out to define the integrity of computationally-predicted relationships and the nature of biological interactions.

In these experiments, AHR was downregulated using a specific silencing (si)RNA specific and the relative expression of genes within the network was measured by RT-PCR in cells treated with BaP to reactivate L1. siRNA targeting the AHR blocked L1 inducibility and modified the expression of other genes within the predicted biological network. These findings established a role for members of the PAS superfamily of proteins in the regulation of retroelement reactivation profiles in mammalian cells. The specificity of these interactions was confirmed in subsequent experiments showing that genetic or pharmacological targeting of the AHR blocks L1 inducibility in multiple cell types [8]. In other computationally-based experiments we found that the connectivity of genes with the discrete network defined by CoD, was not accounted for by physical proximity within the genome and that the network shared multiple components and inputs. More recently, we have turned our attention to elucidation of transcriptional regulatory mechanisms that define physiological connectivity within the L1 network. Earlier we had established a relationship between an AP-1-like responsive element activated by redox stress and the transcriptional activation of L1 in murine cells [5].

Gene transcription is controlled via sequence elements that are recognized and bound by transcription factors and by chromatin modifications at the DNA and histone levels. Transcriptional regulation is often combinatorial in nature and therefore a major goal in studies of genetic regulation is the identification of combinatorial interactions that cooperate in the regulation of gene expression and that constitute a recurrent regulation motif for environmental interference. Work by others established that global hypomethylation of CpG islands leads to L1 reactivation, chromosomal instability and elevated mutation rates and that the E2F/Rb macromolecular complex binds CpG islands to regulate gene expression. Of interest to the regulation of L1 is that the E2F/RB complex associates with histone methyltransferases and histone deacetylases to regulate gene expression. On the basis of these important biological relationships we have hypothesized that L1 reactivation by environmental stress is associated with modulation of DNA and histone methylation and differential recruitment of chromatin modifying complexes onto the L1 DNA template. Preliminary studies support this hypothesis and shown enrichment of L1 DNA amplification in mouse and human cells using E2F1 and E2F4 antisera compared to non-specific antibody. In fact, Rb-deficient cells appear to exhibit exacerbated L1 expression upon chemical stress.

## Conclusion

Studies of L1 biology using toxicogenomics approaches have shed light into the complex biology of retroelements within the human and murine genomes. These studies are helping to bridge the gap by taking advantage of mathematical models to achieve a formal and unified description of biophysical phenomena. Our approach also emphasizes the importance of biological validation of theory and computation and the need for experimentation to rely upon, and be guided by, theory and computation.

The CoD algorithm has been successfully used to define structural and functional determinants of a genetic regulatory network of L1. RNA Pol II and a per homolog were identified computationally as primary attractors within the L1 network, and AHR found to predictably regulate genes within the L1 regulatory network. E2F/Rb complexes may participate in epigenetic regulation of L1 via nucleosomal histone modifications and recruitment of HDACs 1 and 2. As such, L1 reactivation may be due to failure of co-repressor protein recruitment by Rb reflecting loss of histone epigenetic marks and histone acetylation.

Functional genomics approaches can help unravel the complexity of biological systems. The integration of technological advances in the fields of genomics, computational biology and mathematics has led to emergence of systems biology as a means of mapping and managing the complex interactions that govern biological systems and the discovery of novel biomarkers with diagnostic and clinical applications.

## Competing interests

The author declares that they have no competing interests.
